# Type 2 Diabetes Among Filipino American Adults in the Multiethnic Cohort

**DOI:** 10.5888/pcd18.210240

**Published:** 2021-11-24

**Authors:** Phyllis Aira Sheer H. Raquinio, Gertraud Maskarinec, Rica Dela Cruz, Veronica W. Setiawan, Bruce S. Kristal, Lynne R. Wilkens, Loïc Le Marchand

**Affiliations:** 1 University of Hawaii Cancer Center, Honolulu, Hawaii; 2 University of Southern California, Los Angeles, California; 3 Brigham and Women’s Hospital and Harvard Medical School, Boston, Massachusetts

## Abstract

**Introduction:**

Several Asian racial and ethnic groups, including individuals of Filipino ancestry, are at higher risk of developing type 2 diabetes than White individuals, despite their lower body mass index (BMI). This study examined determinants of type 2 diabetes among Filipino American adults in the Multiethnic Cohort Study.

**Methods:**

Participants in Hawaii and Los Angeles completed questionnaires on demographics, diet, and anthropometrics. Generational status was determined according to birthplace of participants and their parents. Based on self-reported data and data on medications, type 2 diabetes status was classified as no, prevalent, or incident. We used polytomous logistic regression, while adjusting for confounders, to obtain odds ratios.

**Results:**

Among 10,681 Multiethnic Cohort Study participants reporting any Filipino ancestry, 57% were 1st-, 17% were 2nd-, and 25% were 3rd-generation Filipino Americans. Overall, 13% and 17% of participants had a prevalent or incident type 2 diabetes diagnosis. Overweight and obesity and the presence of other risk factors increased from the 1st to subsequent generations. First-generation immigrants were less likely to report type 2 diabetes at cohort entry than immigrants of subsequent generations who were born in the US or whose parents were born in the US; only the prevalence of type 2 diabetes was significantly elevated in the 2nd generation compared with the 1st generation.

**Conclusion:**

The results support the hypothesis that Filipino migrants adopt lifestyle factors of the host country and subsequent generations experience higher type 2 diabetes rates due to changes in risk factor patterns.

**Summary**

**What is already known on this topic?**

Filipino American adults have a higher risk of developing type 2 diabetes than White adults, other Asian adults, and residents of the Philippines despite their relatively low body weight.

**What is added by this report?**

Among Filipino American adults, the prevalence of overweight and obesity increased from the first to the 3rd generation, whereas rates of type 2 diabetes were only significantly higher in the 2nd generation than the 1st generation.

**What are the implications for public health practice?**

Overweight and obesity, diet quality, and other lifestyle factors may explain the higher type 2 diabetes rates among 2nd-generation Filipino American adults. Culturally appropriate interventions are needed to reduce lifestyle factors that result in high rates of type 2 diabetes among Filipino immigrants and their descendants.

## Introduction

According to the National Diabetes Statistics Report (1), more than 34 million people in the United States have been diagnosed with type 2 diabetes. Risk factors for type 2 diabetes include excess body weight and physical inactivity (2). People from many ethnic groups (eg, Asian, Pacific Islander, Latino, African American) are at higher risk of developing type 2 diabetes than White people (3). These include individuals of Filipino ancestry, despite having a lower body mass index (BMI) than other ethnic groups (4,5). In the Multiethnic Cohort (MEC) Study, an epidemiologic study of chronic disease risk among more than 200,000 residents of Hawaii and Los Angeles, Filipino American adults had a mean body mass index (BMI) of 23.9 kg/m^2^, compared with 24.6 kg/m^2^ and 27.7 kg/m^2^ in White and Native Hawaiian adults, respectively (6). According to 2017–2018 National Health and Nutrition Examination Survey data, Filipino American adults had the second highest type 2 diabetes prevalence (10.4%) among Asian Americans, following Asian Indians at 12.6%; the rate of type 2 diabetes for Chinese adults was 5.6% (1). A California report showed incidence rates of 14.7 cases per 1,000 person-years for Filipino American adults compared with 7.5 for Japanese adults and 6.5 for Chinese adults; only South Asian adults (17.6), Pacific Islander adults (19.9), and Korean adults (20.3) had higher rates (3). In the MEC, the risk of developing incident type 2 diabetes was 2.5-fold for Filipino American adults compared with White adults and was higher than for all other Asian Pacific Islander adults (6). Moreover, nonobese Filipino American adults were twice as likely to develop type 2 diabetes as nonobese non-Hispanic White adults in a population-based study (7). A geographic comparison showed a greater type 2 diabetes prevalence in San Diego (14.1%) and Hawaii (14.7%) than in the Philippines (11.8%) (8), just as higher rates were reported for multiple American Asian groups (1) in comparison to those in their native country (9).

A high prevalence of risk factors for chronic diseases, including obesity, smoking, and binge drinking, has also been reported for Filipino American people (10). In terms of diet, rice, fruits, vegetables, and fish or meat were reported as typical parts of daily meals (11), and intake of vegetables, fruits, plants, whole grains, and fiber was inversely associated with type 2 diabetes (12). With acculturation, intake of total energy and percentage of calories from fat increased, similar to increases in BMI (13). Although Filipino American people constitute 15.1% of Hawaii’s population and 0.9% of the US population as of 2019 (14), they are understudied because they are commonly aggregated under the racial and ethnic groups of Asian or Pacific Islander (15,16).

Migrant studies comparing risk factors and disease incidence across generations have made important contributions to the understanding of disease etiology, as generational status, shift in risk factors, prevalence, and age at migration have a role in understanding how changes in exposure affect disease risk (17). Selection bias — sometimes called the “healthy immigrant hypothesis,” which states that healthier individuals are more likely to migrate than those in poor health — may underlie the often-seen better health outcomes for 1st-generation immigrants (18,19). In the new country, however, immigrants and their descendants typically experience changes in risk factors (17). As shown for cancer incidence among Japanese (20) and Filipino (21) migrants, risks align more closely to the levels of the host country, often after adoption of behaviors prevalent in the new country. To explore possible reasons for the high type 2 diabetes incidence among Filipino American people despite their relatively low obesity rates, we compared risk factors among 1st generation (born in the Philippines), 2nd generation (born in the US to both parents born in the Philippines), and 3rd generation (born in the US to at least 1 US-born parent) Filipino American adults in the MEC.

## Methods

### Study population

From 1993 to 1996, more than 215,000 men and women aged 45 to 75 years from Hawaii and Los Angeles participated in the MEC (22). The goal of this prospective cohort is to examine diet, lifestyle, and genetic risk factors in relation to cancer and other chronic diseases among individuals of White, African American, Native Hawaiian, Japanese American, and Latino ancestry. Driver’s license files were chosen as the primary sampling frame for recruitment and were supplemented with Medicare files (22). Enrollment targeted the 5 selected ethnic groups using ethnic identifiers, in particular surnames and selected zip codes. As actual ethnicity was not known at the time of the mailing, many persons with ancestries outside the 5 selected groups received and responded to questionnaires.

At cohort entry (22), participants were able to self-report as many ancestries as applicable. Individuals reporting several of mixed ancestry were assigned to 1 of the 5 groups, according to the following priority ranking: African American, Native Hawaiian, Latino, Japanese American, White, and Other. Approximately two-thirds of the Other category were Filipino adults; our final study population included these adults as well as individuals in 1 of the 5 major groups who also reported Filipino ancestry. Participants also reported place of birth for themselves and their parents, which was recoded as the US, the Philippines, and Other. Migration status was assigned according to birthplace of the participants and their parents as 1st, 2nd, or 3rd generation.

### Data collection

A 26-page, self-administered, mailed questionnaire was completed by all participants in English (except Latino participants in California, who were given the choice of an English or Spanish questionnaire); the questionnaire collected information on demographic characteristics, anthropometric characteristics, medical history, and dietary intake. Subsequent questionnaires were mailed approximately every 5 years (Q2 in 1998–2002, Q3 in 2003–2008, Q4 in 2008–2012, and Q5 in 2012–2016) to surviving cohort members and collected updated information on bodyweight and medical history. (Questionnaires can be viewed on the MEC’s website at www.uhcancercenter.org/for-researchers/mec-questionnaires.) All cohort members are followed passively until death through vital records and cancer registries. At baseline, data on years of education (≤12, 13–15, ≥16 y) and smoking status (never, past, current) were collected. Mean time spent in sleep, sedentary, moderate, and vigorous activities on a typical day as reported in the baseline questionnaire were used to compute summary variables for moderate to vigorous activity (<0.5 and ≥0.5 h/d) and sleep duration (<7, 7–8, and >8 h) (22). BMI was computed from self-reported height and weight and categorized into underweight (<18.5 kg/m^2^), normal weight (18.5 to <25.0 kg/m^2^), overweight (25.0 to <30.0 kg/m^2^), and obese (≥30.0 kg/m^2^).

The self-administered survey at cohort entry included a quantitative food frequency questionnaire (QFFQ) with more than 180 food items (23). The unique attributes of the QFFQ included ethnic-specific foods, reliance on a food composition table specific to the MEC, and use of a large recipe database. Although all foods central to a Filipino diet were not listed in the QFFQ, consumption for some (eg, chicken adobo under “roasted, baked, grilled, or stewed chicken”) by other ethnic groups was common enough to include them. Food mixtures were disaggregated into their components, and each ingredient was assigned to the relevant food item. Individual food items and foods from mixed dishes were also classified into food groups based on the My Pyramid Equivalents Database to compute the Healthy Eating Index (HEI)-2010, an indicator of diet quality reflecting the 2010 Dietary Guidelines for Americans, with higher scores indicating better adherence (24). It consists of 12 components, 9 focused on adequacy (total vegetables, greens and beans, total fruit, whole fruit, whole grains, dairy, total protein foods, seafood and plant protein, fatty acid ratio) and 3 focused on moderation (refined grains, salt intake, empty calories [ie, energy from solid fat, added sugars, and alcohol]) (25). Each component is calculated on a density basis per 1,000 kcal. The continuous HEI-2010 scores (26) that range from 0 to 100 (100 equals highest diet quality) were split into tertiles, with tertile 3 indicating the highest diet quality. From the QFFQ, alcohol intake was categorized as less than 1 drink per month, 1 or more drinks per month to less than 1 drink per day, and 1 or more drinks per day.

Information on type 2 diabetes was obtained by self-report in questionnaires at cohort entry and at 4 follow-ups (Q2–Q5). Each questionnaire asked, “Has your doctor ever told you that you had diabetes?” Information on medication for diabetes was collected starting at Q2 and on the biospecimen questionnaire administered as part of a biorepository among 68,740 cohort members established in 2001–2006. To maximize consistency across the study population, we used a self-reported diagnosis or type 2 diabetes medication in at least 1 questionnaire as criteria to define cases, as high validity of self-reports for type 2 diabetes has been demonstrated (27). Participants who reported type 2 diabetes at cohort entry were classified as “prevalent” cases, those who self-reported type 2 diabetes or medication in a follow-up questionnaire as “incident” cases, and all others were classified as “no” type 2 diabetes.

### Statistical analysis

We used SAS version 9.4 (SAS Institute Inc) to analyze all data. After exclusion of cohort members without Filipino ancestry and those with invalid dietary and type 2 diabetes information, the final data set included 10,681 cohort participants. Descriptive analyses were applied to show population characteristics by type 2 diabetes status. We applied multinomial logistic regression using the total study population to compute odds ratios (ORs) and prevalence ORs (PORs) to model more than 2 discrete outcomes (28). To assess the influence of acculturation, migration status was modeled for the 2nd and 3rd versus the 1st generation as the reference group in relation to risk factors. Similarly, we examined the association of type 2 diabetes status (prevalent and incident vs no type 2 diabetes as the reference group) with relevant risk factors (age at cohort entry, sex, BMI, education, smoking status, alcohol intake, physical activity, sleep duration, and HEI-2010 tertiles) that were associated with type 2 diabetes in previous analyses (29,30). Area (Hawaii vs Los Angeles) was not included because the variable did not substantially influence the results. Missing values for covariates were coded as a separate category and included into the statistical models. In addition, the 12 individual HEI-2010 components were examined individually using the same approach both for migrant and type 2 diabetes status.

## Results

Among the 10,681 participants with Filipino ancestry, 70% did not have type 2 diabetes, 13% reported prevalent type 2 diabetes, and 17% were classified as incident type 2 diabetes (Table 1). The percentage of any type 2 diabetes diagnosis was similar in Hawaii and Los Angeles (30% vs 27%). At cohort entry, the mean age of all 3 groups was 57.8 (SD, 8.5) years with no diabetes, 59.7 (SD, 7.9) years with prevalent diabetes, and 55.8 (SD, 7.3) years with incident diabetes. Participants with prevalent type 2 diabetes were diagnosed at a mean age of 60.2 (SD, 8.0) years, and those with incident type 2 diabetes at 67.5 (SD, 7.7) years.

**Table 1:** Characteristics of the Study Population (N = 10,681) at Cohort Entry, Multiethnic Cohort Study^a^

Characteristic	All	No Type 2 Diabetes	Prevalent Type 2 Diabetes	Incident Type 2 Diabetes
**Total**	10,681 (100)	7,530 (70)	1,350 (13)	1,801 (17)
**Area**				
Hawaii	7,281 (68)	5,061 (67)	947 (70)	1,273 (71)
Los Angeles	3,400 (32)	2,469 (33)	403 (30)	528 (29)
**Sex**				
Male	4,875 (46)	3,412 (45)	677 (50)	786 (43)
Female	5,806 (54)	4,118 (55)	673 (50)	1,015 (57)
**Race and ethnicity**				
Filipino	8,152 (76)	5,857 (78)	1,006 (75)	1,289 (72)
Mixed	2,529 (24)	1,673 (22)	344 (25)	512 (28)
**Ethnicity of father**				
Filipino	10,170 (95)	7,153 (95)	1,299 (96)	1,718 (95)
Other	511 (5)	377 (5)	51 (4)	83 (5)
**Ethnicity of mother**				
Filipino	8,888 (83)	6,357 (84)	1,086 (80)	1,445 (80)
Other	1,793 (17)	1,173 (16)	264 (20)	356 (20)
**Migration status^b^**				
1st generation	6,121 (57)	4,510 (60)	673 (50)	938 (52)
2nd generation	1,812 (17)	1,197 (16)	301 (23)	314 (18)
3rd generation	2,659 (25)	1,763 (24)	365 (27)	531 (30)
**Birthplace**				
United States	4,507 (42)	2,983 (40)	672 (50)	852 (47)
Philippines	6,124 (57)	4,513 (60)	673 (50)	938 (52)
Other country^c^	50 (1)	34 (<1)	5 (<1)	11 (1)
**Birthplace of parents**				
Both in United States	694 (6)	470 (6)	83 (6)	141 (8)
One in Philippines	2,186 (20)	1,459 (19)	300 (22)	427 (24)
Both in Philippines	7,750 (73)	5,570 (74)	960 (71)	1,220 (67)
Other country^c^	51 (1)	31 (1)	7 (1)	13 (1)
**Mean (SD) age, y**	57.7 (8.3)	57.8 (8.5)	59.7 (7.9)	55.8 (7.3)
**Mean (SD) age at type 2 diabetes diagnosis, y**	NA	NA	60.2 (8.0)	67.5 (7.7)
**Years of education^b^**				
≤12	4,341 (41)	3,020 (40)	625 (46)	696 (39)
13–15	2,645 (25)	1,847 (25)	331 (25)	467 (26)
≥16	3,587 (34)	2,586 (35)	381 (28)	620 (34)
**Mean (SD) BMI, kg/m^2^**	25.5 (4.5)	24.8 (4.1)	27.2 (5.2)	27.1 (4.5)
**Mean (SD) HEI-2010 score^d^**	63.2 (10.3)	63.0 (10.4)	65.4 (10.1)	62.5 (10.2)
**Mean (SD) total energy intake, kcal/d**	2,535 (1,289)	2,518 (1,279)	2,525 (1,311)	2,613 (1,310)
**Alcohol intake^b^, no. of drinks**				
<1/mo	6,887 (65)	4,713 (63)	990 (73)	1,184 (66)
≥1/mo to <1/d	2,749 (26)	1,996 (27)	276 (20)	477 (26)
≥1/d	1,022 (9)	807 (11)	78 (6)	137 (8)
**Smoking status^b^**				
Never	5,320 (50)	3,818 (51)	598 (44)	904 (50)
Past	3,605 (34)	2,430 (32)	568 (42)	607 (34)
Current	1,616 (15)	1,181 (16)	170 (13)	265 (15)
**Sleep duration, h^b^**				
<7	4,856 (46)	3,356 (45)	643 (48)	860 (48)
7–8	4,870 (46)	3,497 (48)	561 (42)	812 (45)
>8	661 (6)	453 (6)	108 (8)	100 (6)
**Moderate to vigorous physical activity, h/d^b^**				
<0.5	4,560 (43)	3,169 (42)	651 (48)	740 (41)
≥0.5	5,927 (56)	4,222 (56)	668 (50)	1,037 (58)

Abbreviations: BMI, body mass index; HEI, Healthy Eating Index; NA, not applicable.

a Values are expressed as number (%) unless otherwise indicated.

b Missing values: migration status (n = 89), education (n = 108), alcohol intake (n = 23), smoking status (n = 140), sleep duration (n = 294), physical activity (n = 194).

c Other countries include Mexico; Central or South America; Europe; Africa; Cuba or Caribbean Islands; China, Hong Kong, or Taiwan; Japan (includes Okinawa); Korea.

d The Healthy Eating Index (HEI)-2010 is an indicator of diet quality based on the recommendations of the 2010 Dietary Guidelines for Americans, with higher scores indicating better adherence.

Of all participants, 95% reported a father with Filipino ancestry, and 83% reported a mother with Filipino ancestry (Table 1). Fifty-seven percent of participants were born in the Philippines (1st generation), and most others were born in the US (42%). Of the participants’ parents, 73% were both born in the Philippines, 20% of the participants had 1 parent who was born in the Philippines, and 6% were both born in the US. As a result, 17% of participants were classified as 2nd generation and 25% as 3rd generation. The respective proportions of 1st generation participants were lower in Los Angeles than Hawaii (8% vs 58%). Of 1st-generation immigrants, 74% did not have type 2 diabetes, 11% reported prevalent type 2 diabetes, and 15% reported incident type 2 diabetes. Among 2nd- and 3rd-generation Filipino American adults, only 66% had no type 2 diabetes diagnosis and in both groups the proportions of prevalent versus incident cases were similar. Participants with prevalent (27.2 [SD, 5.2] kg/m^2^) and incident (27.1 [SD, 4.5] kg/m^2^) type 2 diabetes had a higher BMI at baseline than those with no type 2 diabetes (24.8 [SD, 4.1] kg/m^2^). The prevalence of overweight and obesity increased from 1st to the 3rd generation (36%, 58%, and 68%).

### Lifestyle factors by generational status

Substantial differences were found in demographics and lifestyle factors among participants by generational status (Table 2). Compared with the 1st generation, 2nd- and 3rd-generation Filipino American adults were less likely to complete 16 or more years of education and were also 2 to 3 times more likely to be past or current smokers, to consume alcohol, and to report 0.5 or more hours per day of moderate to vigorous physical activity. Compared with 1st-generation Filipino American adults, 2nd- and 3rd-generation Filipino American adults were 2 to 3 more times likely to be classified as overweight (PORs of 2.08 and 2.88, respectively) and 5 to 11 times more likely to be classified as obese (PORs of 5.19 and 11.2, respectively). They were also more likely to report high diet quality as indicated by being in the 3rd HEI-2010 tertile, with higher scores for whole grains, dairy, total protein foods, fatty acid ratio, sodium, and refined grains and lower scores on total and whole fruits as well as empty calories (data not shown). In addition, the 2nd generation scored higher on greens and beans and the 3rd generation scored lower on seafood and plant protein, while total vegetable intake was similar for both generations.

**Table 2:** Risk Factors Associated With Migration Status Among Filipino Americans in the Multiethnic Cohort

Characteristic^a^	2nd vs 1st Generation, POR (95% CI)	3rd vs 1st Generation, POR (95% CI)
**Age, y**	1.01 (1.00–1.02)	0.91 (0.90–0.92)
**Sex**		
Male	1 [Reference]	
Female	1.86 (1.62–2.13)	2.38 (2.09–2.72)
**Type 2 diabetes status**		
No	1 [Reference]	
Prevalent	1.25 (1.05–1.48)	1.11 (0.93–1.33)
Incident	1.10 (0.94–1.29)	0.94 (0.81–1.09)
**Years of education**		
<12	1 [Reference]	
12–15	0.57 (0.50–0.66)	0.56 (0.49–0.63)
≥16	0.15 (0.13–0.18)	0.16 (0.14–0.18)
**Smoking status**		
Never	1 [Reference]	
Past	2.27 (1.97–2.61)	2.47 (2.17–2.83)
Current	2.13 (1.78–2.56)	2.99 (2.53–3.51)
**BMI, kg/m^2^**		
<18.5 (Underweight)	0.75 (0.48–1.17)	0.86 (0.54–1.37)
18.5 to <25.0 (Normal weight)	1 [Reference]	
25.0 to <30.0 (Overweight)	2.08 (1.84–2.37)	2.88 (2.55–3.26)
≥30.0 (Obese)	5.19 (4.25–6.34)	11.2 (9.33–13.5)
**Moderate to vigorous physical activity, h/d**		
<0.5	1 [Reference]	
≥0.5	1.39 (1.23–1.56)	1.89 (1.68–2.12)
**Sleep duration, h**		
<7	1.04 (0.93–1.18)	0.88 (0.78–0.99)
7–8	1 [Reference]	
>8	0.90 (0.70–1.16)	1.36 (1.09–1.70)
**Alcohol intake, no. of drinks**		
<1/mo	1 [Reference]	
≥1/mo to <1/d	1.03 (0.90–1.19)	1.29 (1.13–1.48)
≥1/d	1.64 (1.34–2.01)	2.05 (1.69–2.48)
**HEI-2010 tertile^b^**		
1	1 [Reference]	
2	1.19 (1.04–1.36)	1.07 (0.94–1.21)
3	2.21 (1.89–2.57)	2.27 (1.95–2.63)

Abbreviations: BMI, body mass index; HEI, Healthy Eating Index; POR, prevalence odds ratio.

a Values obtained through polytomous logistic regression with adjustment for all variables shown in the table.

b The Healthy Eating Index (HEI)-2010 is an indicator of diet quality based on the recommendations of the 2010 Dietary Guidelines for Americans, with higher scores/tertiles indicating better adherence.

### Predictors of type 2 diabetes status

After full adjustment, only 2nd-generation Filipino American adults were more likely to report prevalent type 2 diabetes at cohort entry (POR = 1.23; 95% CI, 1.04–1.46) than the 1st generation; migration status was not significantly related to incident type 2 diabetes (Table 3). Among lifestyle factors, underweight participants were less likely to report prevalent (POR = 0.52; 95% CI, 0.29–0.95) and incident type 2 diabetes (OR = 0.39; 95% CI, 0.21–0.74). In contrast, overweight was associated with higher rates of prevalent (POR = 1.63; 95% CI, 1.42–1.87) and incident (OR = 2.24; 95% CI, 1.99–2.53) type 2 diabetes as compared with normal-weight participants. Obese participants were nearly 4 times as likely to have prevalent (POR = 3.82; 95% CI, 3.18–4.58) and incident type 2 diabetes (OR = 3.71; 95% CI, 3.13–4.38). Past smokers reported significantly more and those reporting alcohol consumption reported significantly less prevalent type 2 diabetes, and those who had 1 or more drinks per day were also less likely to be diagnosed with incident type 2 diabetes. Getting less than 7 hours of sleep (POR = 1.17; 95% CI, 1.03–1.33) and more than 8 hours of sleep (POR = 1.29; 95% CI, 1.02–1.64) was significantly associated with prevalent type 2 diabetes. Participants in HEI-2010 tertiles 2 (POR = 1.38; 95% CI, 1.20–1.58) and 3 (POR = 1.71; 95% CI, 1.46–2.00) had higher rates of prevalent type 2 diabetes than those in tertile 1; odds of incident type 2 diabetes with respect to HEI-2010 tertile were not significant. For the HEI-2010 components, participants who reported prevalent type 2 diabetes scored higher on total vegetables, greens and beans, whole fruits, whole grains, dairy, total protein foods, fatty acid ratio, and empty calories and lower on sodium. The only component to be associated with incident type 2 diabetes was a higher fatty acid ratio score (data not shown).

**Table 3:** Risk Factors of Diabetes Status Among Filipino Americans (N = 10,681) in the Multiethnic Cohort

Characteristic^a^	Prevalent Type 2 Diabetes vs None, POR (95% CI)	Incident Type 2 Diabetes vs None, OR (95% CI)
**Age, y**	1.03 (1.02–1.03)	0.98 (0.97–0.98)
**Sex**		
Male	1 [Reference]	
Female	0.72 (0.63–0.83)	1.08 (0.95–1.22)
**Migration status**		
1st Generation	1 [Reference]	
2nd Generation	1.23 (1.04–1.46)	1.10 (0.94–1.29)
3rd Generation	1.14 (0.96–1.36)	0.94 (0.81–1.08)
**Years of education**		
<12	1 [Reference]	
12–15	1.02 (0.87–1.19)	1.05 (0.91–1.20)
≥16	0.95 (0.81–1.12)	1.11 (0.97–1.27)
**Smoking status**		
Never	1 [Reference]	
Past	1.34 (1.16–1.55)	1.04 (0.91–1.19)
Current	1.16 (0.95–1.42)	0.97 (0.82–1.14)
**BMI, kg/m^2^**		
<18.5 (Underweight)	0.52 (0.29–0.95)	0.39 (0.21–0.74)
18.5 to <25.0 (Normal weight)	1 [Reference]	
25.0 to <30.0 (Overweight)	1.63 (1.42–1.87)	2.24 (1.99–2.53)
≥30.0 (Obese)	3.82 (3.18–4.58)	3.71 (3.13–4.38)
**Moderate to vigorous physical activity, h/d**		
<0.5	1 [Reference]	
≥0.5	0.77 (0.68–0.88)	0.99 (0.89–1.11)
**Sleep duration, h**		
<7	1.17 (1.03–1.33)	1.07 (0.96–1.20)
7–8	1 [Reference]	
>8	1.29 (1.02–1.64)	0.88 (0.69–1.11)
**Alcohol intake, no. of drinks**		
<1/mo	1 [Reference]	
1/mo to <1/d	0.60 (0.51–0.70)	0.90 (0.79–1.02)
≥1/d	0.41 (0.32–0.53)	0.68 (0.55–0.84)
**HEI-2010 tertile^b^**		
1	1 [Reference]	
2	1.38 (1.20–1.58)	0.94 (0.83–1.06)
3	1.71 (1.46–2.00)	0.86 (0.74–1.01)

Abbreviations: BMI, body mass index; HEI, Healthy Eating Index; POR, prevalence odds ratio.

a Values obtained through polytomous logistic regression with adjustment for all variables.

b The Healthy Eating Index (HEI)-2010 is an indicator of diet quality based on the recommendations of the 2010 Dietary Guidelines for Americans, with higher scores/tertiles indicating better adherence.

## Discussion

First-generation immigrants were less likely to report type 2 diabetes at cohort entry than subsequent generations with self or parents born in the US. Rates of overweight and obesity increased across generations and were the strongest predictor of type 2 diabetes incidence. Participants with prevalent type 2 diabetes had higher scores for most HEI-2010 components (vegetables, greens and beans, protein food, fatty acid, whole grains, dairy, and empty calories) and lower scores for sodium, suggesting that participants with type 2 diabetes possibly adjusted their dietary intake according to recommendations for type 2 diabetes management. The lack of an association between diet quality and incident type 2 diabetes contradicts the MEC report of lower type 2 diabetes incidence associated with high diet quality reported among the entire population (29) but may be due to the relatively small number of cases per group.

The higher rates of type 2 diabetes in the 2nd and 3rd generations than in the 1st may be due to the healthy immigrant hypothesis (19), the younger age at cohort entry, or lack of screening and diagnosis among newly arrived immigrants. Results from a longitudinal study supporting the healthy immigrant hypothesis found that Filipino migrants self-reported significantly better health, less depression, larger hip circumference, and lower waist-to-hip ratio than residents in the Philippines (18). Our results align with the hypothesis that increasing BMI and other lifestyle behaviors may be responsible for the rising type 2 diabetes rates and agrees with findings among women with Filipino ancestry (31), young adults in the Philippines (32), Japanese migrants to the US (33), and a review of worldwide migrant populations (34). Once Filipino immigrants acculturate to the US, they may start to consume a more American diet and eat fewer Filipino foods (13), in addition to adopting other disease risk factors of the host country, as is often described in migration studies (17,21). Indeed, our results indicate that 2nd- and 3rd-generation Filipino American adults were more likely to smoke, consume alcohol, be overweight or obese, and score lower on fruit intake than 1st-generation individuals who had left their home country.

Although only 13% of the participants self-reported type 2 diabetes at cohort entry, 17% developed type 2 diabetes later in life. This finding indicates that, despite the ability of 1st-generation immigrants to migrate from their home country because they are in better health, they or their offspring may still develop adverse health outcomes later in life. We also found that 2nd- and 3rd-generation immigrants were less likely to have a college education than 1st-generation immigrants, even after controlling for other risk factors. Lower educational attainment contributes to increased risk of chronic diseases, including diabetes, and poorer health outcomes (35) and may be a factor at the population level in the development of type 2 diabetes among acculturated US-born Filipino American adults. The high rates of type 2 diabetes among Filipino American adults in this subset of the MEC are similar to those described in previous research, which found that Filipino American adults had rates second only to those of Native Hawaiian adults and Pacific Islander adults in Hawaii (3,5,6,36) and rates that were higher among Filipino adults in the US than in the Philippines (9). 

Our study has several strengths. We had access to information about birthplace and ethnicity of parents among a large study sample of Filipino Americans living in 2 US states. Therefore, it was possible to compare a substantial number of individuals born in the US with those born in the Philippines. The long-term follow-up of more than 20 years, the repeated questionnaires asking for type 2 diabetes status and medication, and the extensive and validated diet questionnaire by QFFQ (23) were additional unique features of the analysis. At the same time, our study also had limitations. Our data were based on self-reports; however, high validity with 84% to 97% specificity, 55% to 80% sensitivity, and more than 92% reliability over time for self-reports of prevalent and incident type 2 diabetes have been demonstrated by other large cohorts (27) and considered sufficiently accurate to allow use in epidemiologic studies (37). Nevertheless, undiagnosed cases due to lack of health care access, possibly more common in the 1st generation, may have been missed. Another limitation was the lack of glucose or HbA_1c_ measures or medical records available for this population. Because the QFFQ was not designed for Filipino individuals, some foods in a typical Filipino diet were not included and may have led to underreporting of total energy intake. This limitation may particularly apply to the 1st and 2nd generation of Filipino American adults, who are more likely to consume a traditional Filipino diet (23). Not all 1st-generation participants may have been fully fluent in English and may have had difficulties in completing the QFFQ. Alternatively, the sample of 1st-generation Filipino American adults in the MEC may not have been representative of this group since those without English skills may not have responded to the survey. Because the physical activity measure was based on recreational and not occupational activities, which are often high among recent immigrants, measurement bias may have occurred. Family history of type 2 diabetes may have influenced the results, but these data were not collected in the MEC. Finally, 24% of participants in this study reported mixed ethnicity and may have adopted food preferences and behaviors that are different from those of the Filipino community.

In conclusion, our results from a prospective cohort suggest that descendants of Filipino immigrants to Hawaii and California have adopted lifestyle factors of the host country, increased their body weight across generations, and developed higher type 2 diabetes rates as a result of these changes in risk factor patterns. Although the prevalence of overweight and obesity increased from the 1st to the 3rd generation, rates of type 2 diabetes were significantly higher only in the 2nd generation compared with the 1st. Therefore, type 2 diabetes incidence may stabilize in the 3rd generation as a result of more acculturation, a finding also reported in a Japanese American cohort (38). Our findings indicate that culturally appropriate interventions are needed to reduce lifestyle factors that result in high rates of type 2 diabetes among Filipino immigrants and their descendants.

## References

[1] US Department of Health and Human Services, Centers for Disease Control and Prevention. National Diabetes Statistics Report 2020. https://www.cdc.gov/diabetes/data/statistics-report/index.html. Accessed August 11, 2021.

[2] Steinbrecher A , Morimoto Y , Heak S , Ollberding NJ , Geller KS , Grandinetti A , The preventable proportion of type 2 diabetes by ethnicity: the Multiethnic Cohort. Ann Epidemiol 2011;21(7):526–35. 10.1016/j.annepidem.2011.03.009 21497517 PMC3109209

[3] Karter AJ , Schillinger D , Adams AS , Moffet HH , Liu J , Adler NE , Elevated rates of diabetes in Pacific Islanders and Asian subgroups: The Diabetes Study of Northern California (DISTANCE). Diabetes Care 2013;36(3):574–9. 10.2337/dc12-0722 23069837 PMC3579366

[4] Lauderdale DS , Rathouz PJ . Body mass index in a US national sample of Asian Americans: effects of nativity, years since immigration and socioeconomic status. Int J Obes Relat Metab Disord 2000;24(9):1188–94. 10.1038/sj.ijo.0801365 11033989

[5] Uchima O , Wu YY , Browne C , Braun KL . Disparities in diabetes prevalence among Native Hawaiians/Other Pacific Islanders and Asians in Hawai’i. Prev Chronic Dis 2019;16:180187. 10.5888/pcd16.180187 30789820 PMC6395081

[6] Maskarinec G , Jacobs S , Morimoto Y , Chock M , Grandinetti A , Kolonel LN . Disparity in diabetes risk across Native Hawaiians and different Asian groups: the Multiethnic Cohort. Asia Pac J Public Health 2015;27(4):375–84. 10.1177/1010539514548757 25164594 PMC4344420

[7] Fuller-Thomson E , Roy A , Tsz-Kit Chan K , Kobayashi KM . Diabetes among non-obese Filipino Americans: findings from a large population-based study. Can J Public Health 2017;108(1):e36–42. 10.17269/CJPH.108.5761 31820422 PMC6972079

[8] Araneta MR , Morton DJ , Lantion-Ang L , Grandinetti A , Lim-Abrahan MA , Chang H , Hyperglycemia and type 2 diabetes among Filipino women in the Philippines, Hawaii, and San Diego. Diabetes Res Clin Pract 2006;71(3):306–12. 10.1016/j.diabres.2005.07.012 16236379 PMC1383725

[9] International Diabetes Federation. IDF Diabetes Atlas 2017. http://www.diabetesatlas.org/. Accessed September 7, 2021.

[10] Ye J , Rust G , Baltrus P , Daniels E . Cardiovascular risk factors among Asian Americans: results from a national health survey. Ann Epidemiol 2009;19(10):718–23. 10.1016/j.annepidem.2009.03.022 19560369 PMC4905692

[11] Johnson-Kozlow M , Matt GE , Rock CL , de la Rosa R , Conway TL , Romero RA . Assessment of dietary intakes of Filipino-Americans: implications for food frequency questionnaire design. J Nutr Educ Behav 2011;43(6):505–10. 10.1016/j.jneb.2010.09.001 21705276 PMC3204150

[12] Kim HS , Park SY , Grandinetti A , Holck PS , Waslien C . Major dietary patterns, ethnicity, and prevalence of type 2 diabetes in rural Hawaii. Nutrition 2008;24(11-12):1065–72. 10.1016/j.nut.2008.05.008 18586461 PMC4351800

[13] Vargas P , Jurado LF . Dietary acculturation among Filipino Americans. Int J Environ Res Public Health 2016;13(1):16. 10.3390/ijerph13010016 PMC4730407 26703646

[14] US Census Bureau. American Community Survey 2019. https://www.census.gov/acs/www/data/data-tables-and-tools/data-profiles/. Accessed September 7, 2021.

[15] Islam NS , Khan S , Kwon S , Jang D , Ro M , Trinh-Shevrin C . Methodological issues in the collection, analysis, and reporting of granular data in Asian American populations: historical challenges and potential solutions. J Health Care Poor Underserved 2010;21(4):1354–81. 21099084 10.1353/hpu.2010.0939 PMC3086449

[16] Shah NS , Kandula NR . Addressing Asian American misrepresentation and underrepresentation in research. Ethn Dis 2020;30(3):513–6. 10.18865/ed.30.3.513 32742157 PMC7360176

[17] Kolonel LN , Wilkens LR . Migrant studies. In: Schottenfeld D, Fraumeni JF, editors. Cancer epidemiology and prevention. 3rd edition. New York (NY): Oxford University Press; 2006. p. 189–201.

[18] de Castro AB , Hing AK , Lee NR , Kabamalan MMM , Llave K , Crespi CM , Cohort profile: The Health of Philippine Emigrants Study (HoPES) to examine the health impacts of international migration from the Philippines to the USA. BMJ Open 2019;9(11):e032966. 10.1136/bmjopen-2019-032966 31727665 PMC6886980

[19] Aldridge RW , Nellums LB , Bartlett S , Barr AL , Patel P , Burns R , Global patterns of mortality in international migrants: a systematic review and meta-analysis. Lancet 2018;392(10164):2553–66. 10.1016/S0140-6736(18)32781-8 30528484 PMC6294735

[20] Haenszel W , Kurihara M . Studies of Japanese migrants — mortality from cancer and other diseases among Japanese in the United States. J Natl Cancer Inst 1968;40(1):43–68. 5635018

[21] Kolonel LN . Cancer incidence among Filipinos in Hawaii and the Philippines. Natl Cancer Inst Monogr 1985;69:93–8. 3834352

[22] Kolonel LN , Henderson BE , Hankin JH , Nomura AMY , Wilkens LR , Pike MC , A multiethnic cohort in Hawaii and Los Angeles: baseline characteristics. Am J Epidemiol 2000;151(4):346–57. 10.1093/oxfordjournals.aje.a010213 10695593 PMC4482109

[23] Stram DO , Hankin JH , Wilkens LR , Pike MC , Monroe KR , Park S , Calibration of the dietary questionnaire for a multiethnic cohort in Hawaii and Los Angeles. Am J Epidemiol 2000;151(4):358–70. 10.1093/oxfordjournals.aje.a010214 10695594 PMC4482461

[24] Liese AD , Krebs-Smith SM , Subar AF , George SM , Harmon BE , Neuhouser ML , The Dietary Patterns Methods Project: synthesis of findings across cohorts and relevance to dietary guidance. J Nutr 2015;145(3):393–402. 10.3945/jn.114.205336 25733454 PMC4336525

[25] Harmon BE , Boushey CJ , Shvetsov YB , Ettienne R , Reedy J , Wilkens LR , Associations of key diet-quality indexes with mortality in the Multiethnic Cohort: The Dietary Patterns Methods Project. Am J Clin Nutr 2015;101(3):587–97. 10.3945/ajcn.114.090688 25733644 PMC4340063

[26] US Department of Health Human Services. 2015–2020 Dietary guidelines for Americans. https://health.gov/our-work/nutrition-physical-activity/dietary-guidelines/previous-dietary-guidelines/2015. Accessed September 7, 2021.

[27] Schneider AL , Pankow JS , Heiss G , Selvin E . Validity and reliability of self-reported diabetes in the Atherosclerosis Risk in Communities Study. Am J Epidemiol 2012;176(8):738–43. 10.1093/aje/kws156 23013620 PMC3571247

[28] Lemeshow S , Hosmer D . Applied logistic regression. New York (NY): Wiley-Interscience Publications; 2000.

[29] Jacobs S , Boushey CJ , Franke AA , Shvetsov YB , Monroe KR , Haiman CA , A priori-defined diet quality indices, biomarkers and risk for type 2 diabetes in five ethnic groups: the Multiethnic Cohort. Br J Nutr 2017;118(4):312–20. 10.1017/S0007114517002033 28875870 PMC5842790

[30] Maskarinec G , Jacobs S , Amshoff Y , Setiawan VW , Shvetsov YB , Franke AA , Sleep duration and incidence of type 2 diabetes: the Multiethnic Cohort. Sleep Health 2018;4(1):27–32. 10.1016/j.sleh.2017.08.008 29332675 PMC5771414

[31] Adair LS , Kuzawa C , McDade T , Carba DB , Borja JB . Seventeen-year changes in body mass index, waist circumference, elevated blood pressure, and diabetes phenotypes in a cohort of Filipino women. Asia Pac J Public Health 2018;30(6):561–71. 10.1177/1010539518800366 30221978 PMC6263034

[32] Uy AB , Jimeno C . Cardiometabolic risk factors leading to diabetes mellitus among the young (YOD) from the 8th Philippine National Nutrition Survey. J ASEAN Fed Endocr Soc 2021;36(1):12–24. 10.15605/jafes.036.01.02 34177083 PMC8214347

[33] Fujimoto WY , Boyko EJ , Hayashi T , Kahn SE , Leonetti DL , McNeely MJ , Risk factors for type 2 diabetes: lessons learned from Japanese Americans in Seattle. J Diabetes Investig 2012;3(3):212–24. 10.1111/j.2040-1124.2012.00195.x 22798980 PMC3393109

[34] Misra A , Ganda OP . Migration and its impact on adiposity and type 2 diabetes. Nutrition 2007;23(9):696–708. 10.1016/j.nut.2007.06.008 17679049

[35] Borrell LN , Dallo FJ , White K . Education and diabetes in a racially and ethnically diverse population. Am J Public Health 2006;96(9):1637–42. 10.2105/AJPH.2005.072884 16873745 PMC1551936

[36] Juarez DT , Davis JW , Brady SK , Chung RS . Prevalence of heart disease and its risk factors related to age in Asians, Pacific Islanders, and Whites in Hawai’i. J Health Care Poor Underserved 2012;23(3):1000–10. 10.1353/hpu.2012.0103 24212153 PMC5677527

[37] Margolis KL , Qi L , Brzyski R , Bonds DE , Howard BV , Kempainen S , ; Women Health Initiative Investigators. Validity of diabetes self-reports in the Women’s Health Initiative: comparison with medication inventories and fasting glucose measurements. Clin Trials 2008;5(3):240–7. 10.1177/1740774508091749 18559413 PMC2757268

[38] Fujimoto WY , Bergstrom RW , Boyko EJ , Chen KW , Kahn SE , Leonetti DL , Preventing diabetes — applying pathophysiological and epidemiological evidence. Br J Nutr 2000;84(Suppl 2):S173–6. 10.1079/096582197388635 11242464

